# Membrane stabilization as a mechanism of the anti-inflammatory activity of methanol extract of garden egg (*Solanum aethiopicum)*

**DOI:** 10.1186/2008-2231-20-76

**Published:** 2012-11-14

**Authors:** Chioma A Anosike, Onyechi Obidoa, Lawrence US Ezeanyika

**Affiliations:** 1 Department of Biochemistry, University of Nigeria, Nsukka, Enugu State, Nigeria; 2 Department of Biochemistry, Kogi State University, Aiymgba, Kogi State, Nigeria

**Keywords:** Inflammation, Leucocyte migration, Vascular permeability, Human red cell membrane

## Abstract

**Background:**

Some observations and reports show that people with high consumption of *Solanum aethiopicum* (African garden egg) have relief in arthritic pains and swelling. We aimed at assessing the effect of methanol extract of *Solanum aethiopicum* in experimentally induced inflammation using leukocyte mobilization and vascular permeability tests in rats and human red blood cell (HRBC) membrane stabilization as studies.

**Methods:**

Twenty five (25) adult Wistar rats of either sex (120 g – 200 g) divided into five groups of five rats each were used for each of the animal models. Groups 2, 3 and 4 were administered varied doses of the extract (100, 200 and 400 mg/kg), while groups 1 (vehicle control) and 5 (treatment control) received normal saline and indomethacin (50 mg/kg) respectively. Vascular permeability was induced by the intra-peritoneal injection of 1 ml of acetic acid and monitored using 0.5 ml intravenous injection of 1% Evans blue solution. Leukocyte mobilization was induced by the intra-peritoneal injection of 0.5 ml of 3% agar suspension in normal saline. Heat and hypotonicity induced heamolysis of HRBC membrane was used to assess membrane stabilization.

**Results:**

The methanol extracts of garden egg significantly and dose dependently reduced (p≤0.05) the acetic acid induced vascular permeability and agar induced leukocyte mobilization in rats. The percentage inhibitions of induced vascular permeability were 21 ± 3.39, 25 ±1.92 and 60 ± 3.81 for the 100, 200 and 400 mg/kg of the extract while the inhibitions of the agar induced leucocyte migration were 23 ± 2.17, 26 ± 1.58 and 32 ± 1.58 for the 100, 200 and 400 mg/kg of the extract respectively. The extract also, at doses of 100, 200, 400, 600 and 800 μg/ml significantly inhibited heat induced lysis of the human red cell membrane with values of 66.46 ± 2.89, 65.14 ± 4.58, 46.53 ± 2.52, 61.88 ± 4.51and 86.67 ± 3.06 respectively.

**Conclusions:**

These results show that methanol extract of *Solanum aethiopicum* has anti-inflammatory properties and can reduce inflammatory injury and tissue damage.

## Background

Inflammation is a complex biological response of vascular tissues to harmful stimuli. It is also a protective attempt by the organism to remove the injurious stimuli and initiate the healing process [1]. At the onset of an inflammation, the cells undergo activation and release inflammatory mediators. These mediators include histamine, serotonin, slow reacting substances of anaphylaxis (SRS-A), prostaglandins and some plasma enzyme systems such as the complement system, the clotting system, the fibrinolytic system and the kinin system [2]. These mediator molecules work collectively to cause increased vasodilatation and permeability of blood vessels. Thus, leading to increased blood flow, exudation of plasma proteins and fluids, and migration of leukocytes, mainly neutrophils, outside the blood vessels into the injured tissues [3]. Inflammation can be classified as either acute or chronic inflammation [1]. Acute inflammation is the initial response of the body to injurious stimuli and is achieved by increased movement of plasma and leukocytes from the blood into the injured tissues. The process of acute inflammation is initiated by cells already present in the tissues. This is characterized by marked vascular changes, including vasodilatation and increased capillary permeability which are induced by the actions of the various inflammatory mediators [4]. Chronic inflammation is a prolonged inflammatory response that leads to a progressive shift in the type of cells present at the site of inflammation and is characterized by simultaneous destruction and healing of the tissues from the inflammatory process [5].

**Figure 1:**
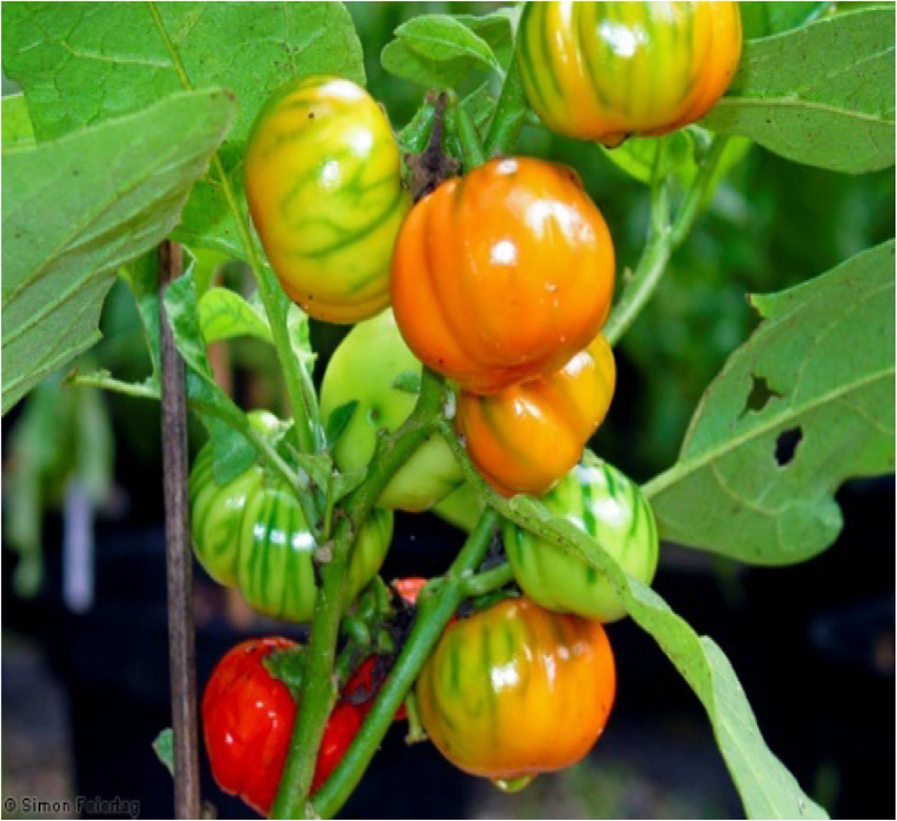
***Solanum aethiopicum *fruits.**

*Solanum aethiopicum* (African garden egg) is a fruiting plant of the genus *Solanum* and family Solanaceae. It is mostly found in Asia and tropical Africa, grows about 2.5m tall, and bears fruits varying in shapes and colours. Some West African species bear edible fruits; they include *S. aethiopicum*, *S. melogena*, *S. macrocarpon*, and *S. muricatum*[6]. The fruits of these species are awakening a growing interest in the speciality and exotic vegetable markets of the world. This is probably associated with the increase in the interest for African cuisine and with African immigration and the potentials of the fruits to improve nutrition [7]. The fruit has various shapes; oval and round, with different colours; white, stripped and green, and turns bright red when ripe (Figure 1). The fruits are usually harvested while still green before the skin becomes thick [8]. These fruits can be eaten fresh without cooking and have a long history of consumption in West Africa. Reports on the pharmacological activity of the garden egg show that it has purgative, sedative, anti-diabetic and anti-ulcerogenic properties [9-11]. The anti-inflammatory effect of the plant on egg albumin-induced oedema and cotton pellet induced granuloma in rats has also been reported [12]. This work was therefore aimed at further assessing the anti-inflammatory properties of the plant extract using experimentally induced vascular permeability and leucocyte mobilization in rats and HRBC membrane stabilization as inflammatory models.

## Methods

### Chemicals

All chemicals used in this study were of analytical grade. They were products of Sigma Aldrich, Germany.

### Plant material

Fresh fruits of garden egg (S*olanum aethiopicum*) were obtained from the Agric Farm of the Faculty of Agricultural Sciences of the University of Nigeria, Nsukka and were identified by Mr. Ugwuozor, a taxonomist of Botany Department, University of Nigeria, Nsukka. A voucher specimen was deposited in the herbarium unit of the Department of Botany, University of Nigeria, Nsukka. The plant was chopped into tiny bits, air-dried for 2 weeks and milled with a mechanical grinder. The ground plant (500 g) was macerated in methanol for 24 hrs, filtered with a white cloth and the filtrate concentrated using a rotary evaporator (IKA, Germany) at an optimum temperature of 40–50°C.

### Animals

Swiss albino mice (22–28 g) and adult Wistar rats (120–200 g) of both sexes obtained from the animal house of the Faculty of Biological Sciences, University of Nigeria, Nsukka were used. They were housed in metal steel cages and acclimatised in the laboratory for seven days before the experiments. They were given free access to water and fed with growers mash (Niger Feeds, Nigeria) bought from the local market. The research was conducted in accordance with the ethical rules and recommendations of the University of Nigeria committee on the care and use of laboratory animals and the revised National Institute of Health Guide for Care and Use of Laboratory Animal (Pub No.85-23, revised 1985).

### Blood sample

Fresh whole blood (3 ml) was collected intravenously from healthy human volunteers into heparinised tubes to prevent coagulation.

### Acute toxicity study

The acute toxicity test of the garden egg extract was carried out by the method of Lorke [13] with some modifications to define the range of lethal dose and safe dose for the extract. Eighteen Swiss albino mice starved of food for 18 hr but allowed access to water were used for the study. They were grouped into six (6) groups of three mice each and treated intraperitoneally (i.p.) with the plant extract at varied dose levels (100, 1000, 1500, 2000, 3000, and 5000 mg/kg). The animals were then observed for nervousness, dullness, in-coordination and or mortality for 24 h. Based on the results of the preliminary toxicity testing, the doses of the extract for further studies were decided to be 100, 200 and 400 mg/kg bodyweight of the rats.

### Phytochemical analysis

The phytochemical analysis of the extract was carried out based on procedures outlined by Trease and Evans [14]. The phytochemicals assayed include alkaloid, glycosides, steroid, terpenoids, flavonoids, tannins, resins and saponinis.

### Leukocyte mobilization test in rats

The effect of the methanol extract of *Solanum aethiopicum* on *in vivo* leukocyte mobilization induced by an inflammatory stimulus was evaluated in albino rats using the method of Rebeiro *et al.,*[15]. Twenty five (25) adult Wistar rats of either sex (120 g – 200 g) divided into five groups of five rats each were used for the test. Groups 2, 3 and 4 were administered varied doses of the extract (100, 200 and 400 mg/kg), while groups 1 (vehicle control) and 5 (treatment control) received normal saline and indomethacin (50 mg/kg) respectively. Three hours after oral administration of the extracts, normal saline or reference drug, each animal in the respective groups (n=5) received intraperitoneal injection (i. p) of 0.5 ml of 3% w/v agar suspension in normal saline. Four hours later, the animals were sacrificed and the peritoneal cavities washed with 5 ml of a 5% solution of EDTA in phosphate buffered saline (PBS). The peritoneal fluid was recovered and both total and differential leukocyte counts (TLC and DLC) were performed on the perfusates using a manual cell counter after staining with Wright’s stain. The percent inhibition of leukoctye migration was calculated using the formula:

$\%\;\text{Leukocyte inhibition}\;\left(\%\;L.I\right)=\left(1-\left(\frac{T}{C}\right)\right)\times \;100$

Where T and C represents the leukocyte count of the treated and control groups respectively.

### Vascular permeability test in rats

The effect of the extract on acetic acid induced vascular permeability was assessed by a modified method of Whittles [16]. Twenty five (25) adult Wistar rats of either sex (120 g – 200 g) divided into five groups of five rats each were used. The animals were fasted for 10 hours prior to the experiment and were then administered with varied doses of the extract and drug as stated above.

Three hours later, each animal was given a 0.5 ml intravenous injection of 1% Evans blue solution. Vascular permeability was induced thirty minutes afterwards, by (i. p) injection of 1 ml of 0.6% acetic acid. The animals were sacrificed 20 minutes later, and their peritoneum washed with 10 ml of normal saline. The recovered peritoneal fluid was centrifuged and the absorbance of the supernatant measured at 610 nm using a spectrophotometer.

### Preparation of erythrocyte suspension

Fresh whole blood (3 ml) collected from healthy volunteers into heparinised tubes was centrifuged at 3000 rpm for 10 min. A volume of normal saline equivalent to that of the supernatant was used to dissolve the red blood pellets. The volume of the dissolved red blood pellets obtained was measured and reconstituted as a 40% v/v suspension with isotonic buffer solution (10 mM sodium phosphate buffer, pH 7.4). The buffer solution contained 0.2 g of NaH_2_PO_4_, 1.15 g of Na_2_HPO_4_ and 9 g of NaCl in 1 litre of distilled water. The reconstituted red blood cells (resuspended supernatant) were used as such.

### Assay of membrane stabilisation

The effects of the garden egg extract on haemolysis of HRBC induced by heat and distilled water was evaluated using the method of Shinde *et al.*[17] with some modifications.

### Heat induced haemolysis

Samples of the extract used were dissolved in isotonic phosphate buffer solution. A set of 5 centrifuge tubes containing respectively, 5 ml graded doses of the extracts (100, 200, 400, 600 and 800 μg/ml) were arranged in quadruplicate sets (4 sets per dose). Two sets of control tubes contained 5 ml of the vehicle and 5 ml of 200 μg/ml of indomethacin respectively. HRBC suspension (0.1 ml) was added to each of the tubes and mixed gently. A pair of the tubes was incubated at 54°C for 20 minutes in a regulated water bath. The other pair was maintained at −10°C in a freezer for 20 minutes. Afterwards, the tubes were centrifuged at 1300 g for 3 min and the haemoglobin content of the supernatant was estimated using Spectronic 21D (Milton Roy) Spectrophotometer at 540 nm. The percent inhibition of haemolysis by the extract was calculated thus:

$\%\;\text{Inhibition of Haemolysis}=1-\left(\frac{OD_{2}-OD_{1}}{OD_{3}-OD_{1}}\right)\times \;100$

Where OD_1_ = absorbance of test sample unheated

OD_2_ = absorbance of test sample heated

OD_3_ = absorbance of control sample heated.

### Hypotonicity induced haemolysis

Samples of the extract used in this test were dissolved in distilled water (hypotonic solution). The hypotonic solution (5 ml) containing graded doses of the extracts (100, 200, 400, 600 and 800 μg/ml) were put into duplicate pairs (per dose) of the centrifuge tubes. Isotonic solution (5 ml) containing graded doses of the extracts (100 – 800 μg/ml) were also put into duplicate pairs (per dose) of the centrifuge tubes. Control tubes contained 5 ml of the vehicle (distilled water) and 5 ml of 200 μg/ml of indomethacin respectively. Erythrocyte suspension (0.1 ml) was added to each of the tubes and mixed gently. The mixtures were incubated for 1 hr at room temperature (37°C), and afterwards, centrifuged for 3 min at 1300 g. Absorbance (OD) of the haemoglobin content of the supernatant was estimated at 540 nm using Spectronic 21D (Milton Roy) spectrophotometer. The percentage heamolysis was calculated by assuming the heamolysis produced in the presence of distilled water as 100%. The percent inhibition of haemolysis by the extract was calculated thus:

$\%\;\text{Inhibition of haemolysis}=1-\left(\frac{OD_{2}-OD_{1}}{OD_{3}-OD_{1}}\right)\times \;100$

Where OD_1_ = absorbance of test sample in isotonic solution

OD_2_ = absorbance of test sample in hypotonic solution

OD_3_ = absorbance of control sample in hypotonic solution

### Statistical analysis

Data obtained were analyzed using SPSS version 15.0 (SPSS Inc. Chicago, IL. USA). All values are expressed as mean ± SD. Data were analysed by one-way ANOVA and difference between means was assessed by Duncan’s new multiple range. P<0.05 was considered statistically significant.

## Results

### Acute toxicity study

Result of acute toxicity study showed that there was no mortality or any significant change in the behaviour of the mice recorded up to the dose of 5000 mg/kg of the plant extract.

### Phytochemical analysis of garden egg extract

The qualitative phytochemical composition of garden egg extract showed relatively high concentration of bioactive compounds such as alkaloids, glycosides, steroids, terpenoids, flavonoids and resins, with saponnins and acidic compounds being in trace amounts, while tannin was not detected (Table 1).

**Table 1:** Result of the phytochemical analysis of garden egg extract

**Phytochemical**	**Garden egg**
Alkaloid	++
Glycosides	+++
Steroids	+++
Terpenoids	+++
Flavonoids	+++
Tannins	-
Acidic compounds	+
Resins	++
Saponnins	+

Bioavailability Key

**- =** not present.

+ = present in low concentration.

++ = present in moderately high concentration.

+++ = present in very high concentration.

### Effect of methanol extract of garden egg on agar induced leukocyte mobilization test in rats

Result obtained from the study showed that the methanol extract of garden egg (*S. aethiopicum*) caused a significant reduction (p≤0.05) in the *in vivo* leukocyte migration induced by agar. The percentage inhibitions of leukocyte migration were 23, 26 and 32% for 100, 200 and 400 mg/kg b.w respectively. Control group treated with indomethacin showed 40% inhibition. The proportion of neutrophils in the perfusate was higher than lymphocytes and other cells in all the groups (Table 2).

**Table 2:** **Effect of methanol extract of garden egg on agar induced *in vivo *leukocyte mobilization**

**Treatment**	**Dose (mg/kg)**	**TLC (X10**^**4**^**)**	**% inhibition**	**Differential Leukocyte mobilization (%)**				
				**Neutrophils**	**Lymphocytes**	**Monocytes**	**Eosinophils**	**Basophils**
Control		94.51 ± 7.93		62.75	34.75	1.0	0.75	0.75
Extract	100	72.62 ± 11.45**	23 ± 2.17	61	35	1.5	1.0	1.5
	200	70.25 ± 12.28**	26 ± 1.58	60	36	1.25	1.5	1.25
	400	64.00 ± 14.78**	32 ± 1.58	57	39	1.25	1.0	1.75
Indomethacin	50	56.7 5 ± 7.89***	40 ± 1.92	60.75	35	1.25	1. 5	1.5

Level of Significance ***= p<0.001, ** = p<0.01, * = p<0.05 Percent inhibition of migration was calculated relative to control.

### Effect of methanol extract of garden egg on acetic acid induced vascular permeability test in rats

Intraperitoneal injection of 0.6% acetic acid caused an increased vasodilation and permeability of the blood vessel of the animals. This was evidenced by the leakage of fluids including Evans blue across the blood vessel epithelial walls into the peritoneal cavity as shown by the bluish colouration of the peritoneal fluid. Data from Table 3 show that the garden egg extract caused a significant and dose dependent reduction (p≤0.05) in vascular permeability. This was evidenced by the reduced blue colouration and thus absorbance values of the peritoneal fluid of the animals treated with the garden egg extract when compared with the control animals.

**Table 3:** Effect of methanol extract of garden egg on acetic acid induced vascular permeability test in rats

**Treatment**	**Dose (mg/kg)**	**Absorbance**	**% inhibition**
Control		0.82 ± 0.12	-
Extract	100	0. 65 ± 0.11*	21 ± 3.39
	200	0.59 ± 0.11*	25 ± 1.92
	400	0.32 ± 0.06**	60 ± 3.81
Indomethacin	50	0.26 ± 0.10**	68 ± 4.08

Level of Significance ***= p<0.001, ** = p<0.01, * = p<0.05 Percent inhibition of migration was calculated relative to control.

### Effect of *S. aethiopicum* extract on heat induced haemolysis of HRBCs

From data shown in Table 4, the garden egg extract at all the doses (100–800 μg/ml) protected the human erythrocyte membrane against lysis induced by heat as is shown by the high percentage inhibitions of haemolysis. The percentage inhibitions of lysis shown by the extract doses were lower than that obtained for 200 μg/ml of indomethacin.

**Table 4:** **Effect of *S. aethiopicum *extract on heat induced haemolysis of HRBCs**

**Treatment**	**Conc. (μg/ml)**	**Mean absorbance ± SD**		**Percentage inhibition of haemolysis**
		**Heated solution**	**Unheated solution**	
Control	**-**	0.67 ± 0.13	0.3 ± 0.01	
**Extract**	**100**	0.25 ± 0.04**	0.4 ± 0.01	66.46 ± 2.89
	**200**	0.28 ± 0.04**	0.08 ± 0.03	65.14 ± 4.58
	**400**	0.42 ± 0. 07**	0.12 ± 0.03	46.53 ± 2.52
	**600**	0.42 ± 0.06**	0.25 ± 0.06	61.88 ± 4.51
	**800**	0.43 ± 0.09**	0.38 ± 0.08	86.67 ± 3.06
Indomethacin	**200**	0.05 ± 0.01***	0.02 ± 0.00	95.32 ± 3.51

Level of Significance ***= p<0.001, ** = p<0.01, * = p<0.05. Percent inhibition of haemolysis was calculated relative to control.

### Effect of *S. aethiopicum* extract on hypotonicity induced haemolysis of HRBCs

Data from Table 5 show that garden egg extracts significantly (p≤0.05) inhibited lysis induced by water. This is shown by the high percentage inhibition of haemolysis (40.8, 53.3 and 50.8) obtained for doses of 400, 600 and 800 μg/ml respectively. The inhibition of haemolysis was found to be dose dependent, increasing with increased concentration of the extract in the medium and was comparable with that obtained for indomethacin (Table 5).

**Table 5:** **Effect of *S. aethiopicum *extract on hypotonicity induced haemolysis of HRBCs**

**Treatment**	**Conc. (μg/ml)**	**Mean absorbance ± SD**		**Percentage inhibition of haemolysis**
		**Hypotonic solution**	**Isotonic solution**	
Control	**-**	0.65 ± 0.11	0.03 ± 0.01	
**Extract**	**100**	0.62 ± 0.1	0.04 ± 0.03	8.8 ± 1.76
	**200**	0.60 ± 0.12	0.07 ± 0.33	11.3 ± 4.16
	**400**	0.48 ± 0.07**	0.18 ± 0.06	40.8 ± 6.75
	**600**	0.5 ± 0.09**	0. 35 ± 0.15	53.3 ± 4.51
	**800**	0.49 ± 0.06**	0.32 ± 0.21	50.8 ± 3.75
Indomethacin	**200**	0.28 ± 0.02**	0.03 ± 0.01	61.47 ± 5.50

Level of Significance ***= p<0.001, ** = p<0.01, * = p<0.05. Percent inhibition of haemolysis was calculated relative to control.

## Discussion

In this study, the efficacy of garden egg extract against induced vascular permeability and infiltration of inflammatory cells to an injured area was carried out using acetic acid induced vascular permeability test and agar induced leukocyte migration. At the onset of an inflammation, the cells undergo activation and release inflammatory mediators that cause vasodilatation and increased permeability of blood vessels leading to the exudation of plasma proteins and fluids into the tissues; the vessel walls become engorged and dilated, allowing large numbers of neutrophils to extravasate and appear within the junctional epithelium and underlying connective tissue spaces [3]. In this study, intraperitoneal injection of 0.6% acetic acid caused an increased dilation and permeability of the blood vessels of the animals which was evidenced by the increased leakage of fluids, including Evans blue across the blood vessel epithelial walls. Administration of the garden egg extract caused a significant and dose dependent reduction in vascular permeability of the extract treated animals.

The extract also significantly (p≤0.05) reduced the migration of leukocytes in response to agar induced inflammatory stimulus. A number of different cells are recruited into an inflammatory area. These cells are responsible for the inactivation and removal of invading infectious agents and damaged tissues. Chemotactic movement of leukocytes towards the foreign body is the first and most important step in phagocytosis [18]. Leukocytes are found in tissues during acute inflammatory process and in the superficial surface aspects of a lesion during subacute or chronic inflammation. They function as phagocytes of bacteria, fungi and viruses, and detoxifiers of toxic proteins that may result from allergic reactions and cellular injury [19]. Eosinophils and basophils are predominant when inflammation is initiated by immediate allergic reactions or parasites [20]. In our result, neutrophils which engulf and eliminate microorganism were the most mobilized leukocytes. Generally, neutrophils are the first line of defense of the immunological system against pathogens. However, in an inflammatory disease such as rheumatoid arthritis, they represent a potential cause of tissue damage [21]. The interaction of recruited neutrophils in the site of inflammation with resident cells, local inflammatory mediators, and extracellular matrix may lead to the production of several other mediators, including cytokines, degrading enzymes, oxygen and nitrogen species that may further amplify the inflammatory response and injure surrounding tissues [22]. Increased infiltration and adhesion of leukocytes to endothelial cells, thus, increases the inflammatory process. The inhibition of leukocyte migration by garden egg extract therefore shows that garden egg could alter the action of the endogenous factors that are involved in the migration of these cells to the site of inflammation, thereby reducing the inflammatory process.

Garden egg extract at concentrations of 100–800 μg/ml protected the human erythrocyte membrane against lysis induced by hypotonic solution and heat. During inflammation, there are lyses of lysosomes which release their component enzymes that produce a variety of disorders. Non-steroidal anti-inflammatory drugs (NSAIDs) exert their beneficial effects by either inhibiting the release of lysosomal enzymes or by stabilizing the lysosomal membranes [23]. Exposure of red blood cells (RBCs) to injurious substances such as hypotonic medium, heat, methyl salicylate or phenylhydrazine results in the lysis of the membranes, accompanied by haemolysis and oxidation of haemoglobin [24]. Since human red blood cell (HRBC) membranes are similar to lysosomal membrane components [23], the inhibition of hypotonicity and heat induced red blood cell membrane lysis was taken as a measure of the mechanism of anti-inflammatory activity of garden egg extract. The haemolytic effect of hypotonic solution is related to excessive accumulation of fluid within the cell resulting in the rupturing of its membrane. Injury to red cell membrane will render the cell more susceptible to secondary damage through free radical induced lipid peroxidation [25]. Membrane stabilization leads to the prevention of leakage of serum protein and fluids into the tissues during a period of increased permeability caused by inflammatory mediators [3]. Garden egg extract perhaps stabilized the red blood cell membrane by preventing the release of lytic enzymes and active mediators of inflammation.

Phytochemical results of this study showed that garden egg is abundantly rich in flavonoids, alkaloids, steroids and terpenoids. These phytochemicals occur in various parts of the plant. Many reports have shown that plant flavonoids possess potent anti-inflammatory and anti-oxidant properties [26-28]. Their anti-inflammatory activities are probably due to their inhibitory effect on enzymes involved in the production of the chemical mediators of inflammation and metabolism of arachidonic acid [29,30].

## Conclusion

This study therefore shows that garden egg has modulatory effect on the vascular changes that occur during acute inflammation. Many anti-inflammatory plants and agents modify inflammatory responses by accelerating the destruction or antagonising the action of the mediators of inflammatory reaction. Foods and fruits rich in flavonoids and other phenolic compounds have been associated with decreased risk of developing inflammatory and other related diseases [31,32]. These reports, thus, suggest that the flavonoids in garden egg plant might be a major anti-inflammatory constituent in the plant.

We conclude that this study has shown that the methanolic extract of the fruits of garden egg (S*olanum aethiopicum*) have anti-inflammatory activity in the models studied.

## Competing interests

The author(s) declare that they have no competing interests.

## Authors’ contributions

OO conceived of the study, and participated in its design and coordination. LUS participated in the coordination of the study. CA carried out the experimental studies and drafted the manuscript. All authors read and approved the final manuscript.
